# The South London and Maudsley NHS Foundation Trust Biomedical Research Centre (SLAM BRC) case register: development and descriptive data

**DOI:** 10.1186/1471-244X-9-51

**Published:** 2009-08-12

**Authors:** Robert Stewart, Mishael Soremekun, Gayan Perera, Matthew Broadbent, Felicity Callard, Mike Denis, Matthew Hotopf, Graham Thornicroft, Simon Lovestone

**Affiliations:** 1 King's College London (Institute of Psychiatry), London, UK; 2 South London and Maudsley NHS Foundation Trust, London, UK

## Abstract

**Background:**

Case registers have been used extensively in mental health research. Recent developments in electronic medical records, and in computer software to search and analyse these in anonymised format, have the potential to revolutionise this research tool.

**Methods:**

We describe the development of the South London and Maudsley NHS Foundation Trust (SLAM) Biomedical Research Centre (BRC) Case Register Interactive Search tool (CRIS) which allows research-accessible datasets to be derived from SLAM, the largest provider of secondary mental healthcare in Europe. All clinical data, including free text, are available for analysis in the form of anonymised datasets. Development involved both the building of the system and setting in place the necessary security (with both functional and procedural elements).

**Results:**

Descriptive data are presented for the Register database as of October 2008. The database at that point included 122,440 cases, 35,396 of whom were receiving active case management under the Care Programme Approach. In terms of gender and ethnicity, the database was reasonably representative of the source population. The most common assigned primary diagnoses were within the ICD mood disorders (n = 12,756) category followed by schizophrenia and related disorders (8158), substance misuse (7749), neuroses (7105) and organic disorders (6414).

**Conclusion:**

The SLAM BRC Case Register represents a 'new generation' of this research design, built on a long-running system of fully electronic clinical records and allowing in-depth secondary analysis of both numerical, string and free text data, whilst preserving anonymity through technical and procedural safeguards.

## Introduction

Case registers have distinct advantages in their ability to provide information on specific disorders that is readily obtainable and available for meaningful analysis. Particular indications for a case register approach are when the disorders in question are rare (i.e. cannot be practically screened for in community populations) and 'high penetrance' (i.e. secondary care samples can be assumed to be representative of total community cases). As with other epidemiological research methods, their focus is on investigating the determinants of health states in specified populations. Distinct features are that the 'specified populations' all have a given disorder or level of service contact, and the 'health states' most often concern the presentation, features, course and outcome of a given disorder rather than risk of the disorder *per se*. As the 'data collection' is systematic and prospective, case registers are able to produce a powerful, longitudinal and comprehensive impression of treated morbidity in any distinctive region. Perhaps the broadest definition of a psychiatric case register is that it is 'a patient-centred longitudinal record of contacts with a defined set of psychiatric services originating from a defined population' [1]. In a UK context, greater health service evaluation, increased research into the effectiveness of individual- and service-level interventions, and analyses of pharmaco-epidemiological data, are integral to the government's Connecting for Health Research Capability Programme . Within mental health, this has created an imperative for case registers that will be able to meet these goals.

Historically, medical records of some kind have always been kept and, in keeping with the tradition of careful methodical scientific observation, these have frequently been developed into disease registers, through which the incidence, course and health service use of specified diseases can be monitored and investigated. In the context of changing social, political, professional and technological factors, a large number of psychiatric registers were constructed throughout the 20^th ^century. However, owing to the expense of maintenance (often carried out manually), the limited information available (relying on data sheets completed by clinicians in addition to their routine workload), the practical difficulties in monitoring data quality and limited funding, many programmes closed in the mid to late 1980s and only a few international registers still exist.

Rapid technological advances and other developments over the last decade have led to new possibilities for case register development. With electronic clinical records increasingly complementing handwritten notes, large volumes of clinical information are contained in an electronic format. Because of this, there are now national case registers worldwide in Israel, Denmark and New Zealand which cover populations from approximately four to six million [2-4]. Whilst national registers are valuable, in that they provide inclusive data on a very large scale, they are most commonly restricted to hospitalised patients [2,3] (except the Danish register which now includes outpatient data [4]). Regional registers in Europe such as the Verona psychiatric case register which covers a population of 80,800 [5] and the Groningen psychiatric case register in the Netherlands which covers a region of approximately 460,000 [6] are more likely to suffer with problems due to internal migration (the numbers of which tend to be higher than migration levels on a national level), but their strength lies in their ability to cover all types of psychiatric services within that area, thereby providing a more comprehensive picture of mental health than afforded by national registers. However, to our knowledge, no case register to date has attempted to capture data from the full clinical record, something which has only become feasible recently with the advent of fully electronic medical records systems.

As medical records become increasingly electronic in nature, the possibility for case registers collected as part of routine health care has therefore become a tangible possibility. However, there remain several significant hurdles to overcome. First, the datasets involved are not in a form accessible to researchers or the statistical programs with which they are most familiar and a high level of technical expertise would be required for even the simplest analysis. Second, a large amount of potentially the most valuable information is contained within free text rather than in checkbox format. Third, there are important concerns about data protection and both technical and procedural issues to consider in generating data which is adequately anonymised at the point of analysis – particularly demanding where free text analysis is envisaged.

In this manuscript we describe the development of a large regional case register which potentially provides a template for addressing these challenges. With records of over 120,000 individual cases representing the total secondary care use of mental health services for a population of 1.1 million, we believe that the recently developed SLAM BRC Case Register is the largest regional register in Europe. The emergence of this psychiatric case register is the result of a collaboration between the South London and Maudsley NHS Foundation Trust and the Institute of Psychiatry at King's College London, and was made possible through a specialist mental health Biomedical Research Centre (BRC) award to the two organisations from the UK government's National Institute for Health Research (NIHR). The development of the case register has paid as close attention to the issues surrounding privacy and security as to the technical challenges and, throughout, has followed the principle of 'consent or anonymise'. This principle follows UK case law interpretation of the European Data Directive (the 'Source Informatics' ruling) which establishes that the use of data for purposes other than that for which it was created does not require consent by the providers of the data, provided that the data are anonymous [7].

## Methods

### The South London and Maudsley NHS Foundation Trust

The South London and Maudsley Foundation NHS Trust (SLAM), whose case records provide the source data for the SLAM BRC Case Register, is, to the best of our knowledge, the largest mental health care provider in Europe, serving an aggregate population of 1.1 million over four London boroughs (Croydon, Lambeth, Lewisham, Southwark) as well as providing a number of specialist national services. Descriptive data for the four boroughs from the national census in 2001 are summarised in Table 1 and compared with those for London and England. Compared with England as a whole, London contains a higher proportion of young adults and lower proportions of middle aged and older adults, with larger proportions reporting higher education levels and higher status occupations. People from minority ethnic groups also form substantially higher proportions of the population in London compared to England. The SLAM catchment boroughs do not differ substantially from London as a whole in terms of age, gender, education and socioeconomic status. Overall proportions from minority ethnic groups are similar to London as a whole but the composition is different, with higher proportions with self-assigned Black ethnicity and lower proportions Asian. All four boroughs show similarly high levels of population mobility, together accounting for approximately one fifth of that of London overall.

**Table 1:** Descriptive statistics (2001 Census data) for the four London boroughs served by SLAM and comparisons with London and England statistics

	Borough				London	England
			
	Lambeth	Croydon	Lewisham	Southwark		
Total population	266,169	330,587	248,922	244,866	7,172,091	49,138,831
Age (%)						
0–19	23.3	26.8	25.7	24.8	24.9	25.1
20–29	22.2	13.3	17.1	19.6	17.0	12.7
30–44	29.2	25.1	28.2	28.3	25.7	22.6
45–74	21.1	28.7	23.7	22.5	26.5	32.1
75+	4.2	6.1	5.3	4.8	5.9	7.5
Gender (%)						
Male	49.3	48.1	48.2	48.9	48.4	48.7
Female	50.7	51.9	51.8	51.1	51.6	51.3
Education* (%)						
No qualifications	20.1	22.9	24.2	24.4	23.7	28.9
Level 2 (5 or more GCSEs at A-C)	14.0	21.4	17.4	14.6	17.1	19.4
Level 3 (2 or more A levels)	9.8	9.3	9.1	10.0	9.8	8.3
Level 4/5 (First degree or higher)	40.9	23.6	29.4	34.8	31.0	19.9
Self-assigned ethnicity (%)						
White	62.4	70.2	65.9	63.0	67.5	90.9
Mixed	4.8	3.7	4.2	3.7	3.6	1.3
Asian or Asian British	4.6	11.3	3.8	4.1	13.6	4.6
Black or Black British	25.8	13.3	23.4	25.9	12.3	2.3
Other	2.5	1.5	2.7	3.3	3.0	0.9
Socio-economic status* (%)						
High managerial/professional	14.0	10.1	9.8	11.7	12.1	8.6
Lower managerial/intermediate	34.1	36.0	33.6	29.3	32.5	28.2
Small employers/lower supervisory	9.8	12.6	11.2	9.9	11.4	14.1
Routine/semi-routine jobs	14.6	15.3	16.2	16.3	14.8	20.7
Never worked/unemployed	6.9	4.7	6.5	7.2	6.0	3.7
Full time students	9.2	6.8	9.4	12.6	9.0	7.0
Migration from mid-2006 to mid-2007 (approximates in thousands)						
Inflow	23.5	18.7	18.4	19.8	167.0	511.0**
Outflow	28.9	20.9	21.1	24.1	284.4	299.0**
Balance	-5.5	-2.3	-2.8	-4.3	-81.5	+212.0**

*Data restricted to persons aged 16–74 years

**2007 data

SLAM provides one of the most extensive portfolios of mental health services in the UK. It supplies a wide range of multidisciplinary and integrated specialist services across General Adult, Child and Adolescent, Forensic, Older Adults, Learning Disabilities and Addiction sections with close to 4500 members of staff. Numbers of active and inactive cases within specified directorates are displayed in Table 2.

**Table 2:** Summary statistics active and inactive cases by Directorate within SLAM (Nov 2008)

Directorate	Number		
	Active	Inactive†	Total
Child and Adolescent Mental Health Services	5857	11,452	17,309
Adult Mental Health	14,932	58,462	73,394
Mental Health of Older Adults	3502	9948	13,450
Forensic	661	378	1039
Addictions	4151	4691	8842
Learning Disabilities	954	841	1795
Specialist national services	5439	5031	10,470

Total	35,496	90,803	126,299

†Inactive: those not currently receiving treatment and who have been discharged from all services

The Trust includes the Maudsley and Bethlem Royal Hospitals whose roles in mental health service developments have been substantial and longstanding, the Bethlem Hospital dating as far back as 1247. Its medical school, founded in the early 1900s, and now known as the Institute of Psychiatry at King's College London, is a leading international centre for mental health research and teaching. SLAM has championed the use of electronic medical records (EMR) for almost a decade and clinical services are now effectively paperless. Our challenge was to create a research interface that would interrogate the data available and that could return datasets appropriate for research use and effectively anonymised at the research interface.

### The Patient Journey System

The SLAM Patient Journey System (PJS) is a bespoke, single, integrated electronic clinical record used across all Trust services. It was designed primarily to support the recording and sharing of clinical information, whilst producing relevant management and national reports as a natural by-product. PJS was implemented in SLAM between October 2005 to October 2006, replacing a number of independent clinical and administrative information systems used in the Trust at the time, including hard copy case notes, a community-based electronic clinical record and Care Programme Approach system, two separate inpatient Patient Administration Systems (PAS), and Adult Mental Health and Addictions service administrative systems. At implementation, all patient-based information for patients seen from 1999 onwards was migrated into PJS from electronic legacy systems.

PJS is a comprehensive record of all clinical information recorded throughout patients' journeys through Trust services, including demographic and contact information, dates and other details of referrals and transfers, detailed clinical assessments, care plans and medication, clinical activity and reviews. Imaging and laboratory data are not, to date, recorded here. The record is used and maintained by multi-disciplinary professionals and consists of both structured data (such as dates, integers and pick-lists) and unstructured free text (including written assessments, progress notes and correspondence). PJS includes specific assessments such as structured physical health assessments, cognitive function (MMSE), and outcome measures such as the Health of the Nation Outcome Scales.

### The SLAM BRC Case Register Interactive Search (CRIS)

**Figure 1:**
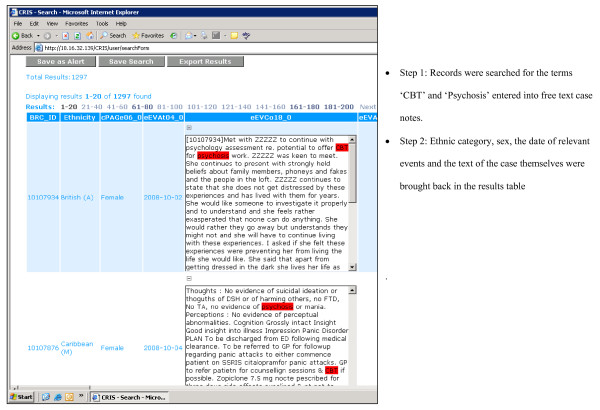
**Screen shot of CRIS results table**.

In terms of core functionality, the CRIS system allows researchers to search against any combination of structured fields (date, numerical etc.) and unstructured fields (user-defined text strings) from PJS records. The tool 'hits' relevant records based on search terms (such as a particular coded diagnosis and/or a particular text string in a clinical assessment). Users then specify the precise fields they want returned, which may be the same as or different from those they searched against. Results are returned in spreadsheet format and are exportable as CSV files for further analysis. An example of a search return is displayed in Figure 1.

**Figure 2:**
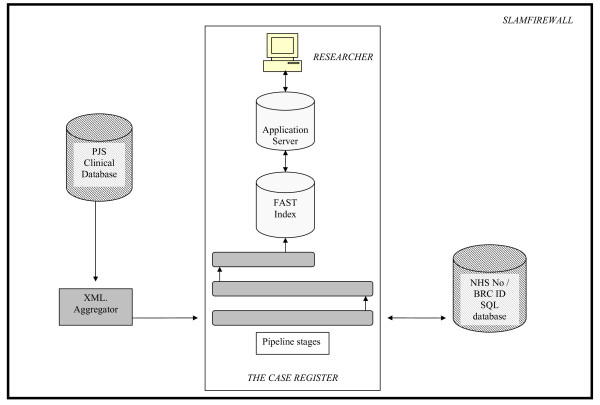
**CRIS technical architecture**.

In terms of technical architecture, CRIS was built in partnership with BearingPoint™ and uses FAST™ search technology. Source PJS clinical information is currently held in Lotus Notes. To build the FAST searchable index these data need to be converted to xml. A bespoke device termed the 'xml. aggregator' was created to pull Notes data from PJS and construct an xml. copy. During the construction process the xml. aggregator also reconstructs a family hierarchy for each record, including all the documents and fields in the record, and cleans out exceptional data anomalies, such as incorrectly formatted data. Once the cleaned xml. version of PJS has been created the data pass through the FAST pipeline stages, which have been configured to meet the anonymisation requirements. At record level, any combinations that match entries in structured name, date of birth and address fields in PJS are masked (replaced with ZZZZZ, as displayed in Figure 1) in the searchable index. In addition, an anonymised BRC identifier is constructed from the service user's NHS number, which is then excluded from the searchable index. NHS numbers are unique to all individuals in the UK and are the primary identifier for all state provided healthcare (both primary and secondary care). They therefore provided the logical solution for avoiding patient duplication and as a source for a unique identifier that will link potentially multiple care episodes. The algorithm for creating BRC identifiers from NHS number is hidden and cannot be accessed by front line researchers. A Java front-end enables users to construct search queries, which are converted to FAST Query Language (FQL) to interrogate the index. The technical architecture behind the CRIS system is summarised in Figure 2.

### The security model

The development of CRIS demanded that due attention be given to legal and ethical considerations attendant upon the use of confidential health data. The IOP/SLAM BRC Stakeholder Participation Theme took a central role here, given that its remit is to ensure that the participation of two key stakeholder groups, service users and carers (along with other groups who are frequently under-represented in research on grounds of gender, age, culture and ethnicity), are at the heart of the BRC's research work. A member of the Stakeholder Participation Theme, herself a mental health service user, chaired a time-limited working group that developed security and confidentiality procedures for the case register (this group also included the Trust Caldicott Guardian [the senior person in UK NHS Trusts responsible for protecting the confidentiality of patient and service-user information and enabling appropriate information sharing], the ex-chair of a Research Ethics Committee, and the child protection lead within the Trust).

Deliberation over how to develop both technical and procedural frameworks that would adequately address the legal and ethical issues raised by the case register took place in the context of lively discussion at a national level over the use of confidential health data. There is growing recognition, both within England and internationally, of the importance and urgency of establishing robust policies, standards and best practices governing the sharing of data in general, and the secondary use of anonymised health data in particular [8].

While recent consultations in the UK [9] have indicated that members of the public in principle approve of the use of databases for biomedical research, they have also indicated that the public wishes to know more about the research process, why certain data are required, how they are stored and interpreted, and who has access to them. In addition, these consultations have pointed to some concerns about the use of 'sensitive' data – including those relating to mental health diagnoses – that carry the potential for stigmatisation. However, relatively little is currently known about what those with psychiatric, neurodegenerative and/or substance use diagnoses themselves think about research databases comprising clinical data [10]. In light of this national context, it was clear that developing frameworks to ensure the ethical and secure use of CRIS demanded a focus on three distinct, though overlapping domains:

**1. Technical measures **How ought the technical (i.e. programming) components of the case register best be designed to protect the confidentiality and anonymity of clinical data?

**2. Procedural measures: **Which procedures and mechanisms are necessary to regulate access to and use of the case register, and to ensure that governance of the register gains and maintains the trust of stakeholders?

**3. Engagement with stakeholders **How best to engage stakeholders (who include service users, carers, health professionals, service support staff, as well as members of the public) in discussions about the case register's contributions to mental health research, and to disseminate information regarding the case register to service users and their carers/families?

A summary of the mechanisms and procedures that were developed to respond to the challenges raised in each of these three domains follows.

#### 1. Technical measures

It was critical to develop robust measures to ensure that important epidemiological data are included in CRIS (e.g. age, gender, ethnicity, broad locality of residence) without compromising the anonymity of service users and of carers/family members. Various data are therefore truncated (e.g. only the first half of the UK postcode, and only the month and year of birth are included). One of the key research strengths of the Case Register – the capacity to interrogate free text as well as structured data – raises particular challenges for anonymisation, given the complexity and extent of free text in the clinical database (PJS). While it is apposite to recall Kalra and colleagues' reminder that 'it is ... wise to consider anonymised data as if there is still some risk of re-identification and disclosure' [8], all efforts were made when developing CRIS's programme architecture to ensure that anonymisation is robust throughout the entirety of each service user's record.

#### 2. Procedural measures

Procedural measures were required both in relation to the initial launch of the Case Register (e.g. mechanisms to ensure that all data are held securely within the Trust firewall, and to control access to the case register) and as regards its ongoing use, monitoring and development. Particular attention has been paid to upholding the scientific rigour of research that interrogates data from the case register. A committee was formed to provide operational oversight of the Case Register, as well as to ensure that any study intending to use the register has scientific value and has taken precautions to diminish the likelihood of any searches compromising confidentiality (e.g. by returning details, from individual or combinations of variables, of rare and therefore potentially identifiable groups of individuals). This Oversight Committee reviews all applications to use the Case Register and also aims to provide practical advice to researchers on how best to navigate and manipulate the complex and extensive data.

Ethics approval was sought for the research use of anonymised databases derived using CRIS, and was granted by an independent Research Ethics Committee ('Oxfordshire C') that is 'flagged' to indicate expertise in research databases. The security model (that comprises both technical and procedural elements) was approved by the Trust Caldicott Guardian and signed off by the Trust Executive who are also responsible for research governance and to whom the Oversight Committee reports. The Oversight Committee is therefore responsible for considering projects using the Case Register for secondary analysis of anonymised data and ensuring that these are compliant with approval criteria as well as being an appropriate use of the data. The Committee is chaired by a mental health service user and member of the SLAM BRC Stakeholder Participation theme, and the Committee has representation from the SLAM Research Ethics Committee, the SLAM Caldicott Guardian, and the SLAM BRC Child and Adolescent Mental Health research theme, in addition to the Case Register Academic Lead and Case Register Project Manager. Researchers wishing to use the Case Register are required to submit a Project Approval Form which asks for a summary of the purpose of the proposed project and the nature of the data required for analysis (so that searches can be audited). No cost is levied for use of the database.

This manuscript focuses on the use of the Case Register for research purposes. However, it is also important to point out that it is also used for Trust audits (i.e. to facilitate projects approved by statutory audit committees). As well as the functions mentioned above, the Oversight Committee is responsible for checking that proposed audits have received appropriate approval and to ensure that there is no unauthorised or unprincipled use including that of data identifying staff members (which are not anonymised).

#### 3. Engagement with stakeholders

A communications plan has been developed to inform the whole Trust community (service users, carers, clinicians and other Trust staff) about the existence, potential benefits and proposed uses of the Case Register. This plan will respond to the findings of the recent consultations about medical research databases cited above through specifically discussing how health research proceeds, why certain data are required, and how they are stored and analysed. The plan has been shaped through consultation with the BRC's service user advisory group. In addition, the Stakeholder Participation Theme of the BRC is interrogating the ethical, societal and practical issues raised by the increased focus on electronic medical records. This research will inform the ongoing development of the case register and contribute to wider discussions concerning secondary uses of health data. The Appendix summarises the mechanisms and procedures that have been developed to address the legal and ethical issues pertaining to the use of confidential health data within the case register. Finally, although under UK law consent is not required for use of anonymised information, the Case Register has the technical capacity to allow opt out by anyone who objects to their data being used for research purposes (i.e. rendering their clinical data inaccessible to the search functions). This option is available in the Case Register. The role of the Oversight Committee includes ensuring that the use of anonymised clinical data for research is widely publicised among service users, carers and Trust staff, involving an ongoing information dissemination plan using different media including Trust information leaflets, public meetings and websites.

## Results

### Summary statistics

Table 3 shows summary statistics of the SLAM BRC Case Register from a search carried out in October 2008. The largest age group of cases lies within the 20 to 40 year age range. There are also approximately equal numbers of males and females represented in the register. The distribution of ethnic groups is broadly consistent with Census distributions in the boroughs served with only marginally fewer identifying themselves as other than British. However, it should be borne in mind that, while both the Census and NHS records aim to collect self-assigned ethnicity, the lists of available categories are different; the two are not therefore directly comparable. The present number of active patients (defined as those who have an active Care Programme Approach on the Register), is approximately 35,000.

**Table 3:** Descriptive summary of SLAM case register records*

	Number (%)
Total	122,440
Age (years)	
≤ 20	20,274 (16.6)
21–40	43,610 (35.6)
41–60	36,305 (29.7)
61–80	12,881 (10.5)
>80 yrs	9370 (7.7)
Gender	
Male	60,833 (49.8)
Female	61,342 (50.2)
Self-assigned ethnicity (full breakdown)**	
British	43,909 (57.8)
Irish	2343 (3.0)
Any other White background	6653 (8.6)
Mixed: White and Black African	354 (0.5)
Mixed: White and Asian	254 (0.3)
Mixed: Any other mixed background	418 (0.5)
Indian	1041 (1.3)
Pakistani	420 (0.5)
Bangladeshi	355 (0.5)
Any other Asian background	1483 (1.9)
Caribbean	5260 (6.8)
African	5360 (6.9)
Any other Black background	6236 (8.0)
Chinese	190 (0.2)
Any other ethnic group	3398 (4.4)
Self-assigned ethnicity (amalgamated)**	
British	52,905 (68.1)
Mixed	1026 (1.3)
Indian, Pakistani, Bangladeshi or 'other Asian'	3299 (4.2)
Caribbean, African or any 'other Black'	16,856 (21.7)
Other	3588 (4.6)
Number with active Care Programme Approach***	35,396 (28.9)

*Data ascertained on 20/10/08

**Recorded for 77,674 (63.4%)

***Implying active care management and ongoing service contact

### Diagnoses

A description of numbers of cases by primary (ICD-10) diagnosis, again from a search in October 2008, is provided in Table 4 for the 65% of all cases where this information was available. In SLAM, diagnoses are generally assigned by members of the clinical team primarily responsible for the patient concerned with team discussion as required. A choice is made from a full list of ICD codes appearing in a checkbox on PJS, and proportions of cases with an assigned diagnosis has been a key factor monitored by the Trust and its funders for the last 2–3 years. Multiple diagnoses may be recorded but, for simplicity, only data on primary diagnoses are displayed here. Of mental disorder diagnoses, the most commonly assigned are mood disorders followed by schizophrenia and related disorders, substance misuse, neuroses and organic disorders.

**Table 4:** Descriptive data on recorded primary diagnoses in the SLAM case register

Assigned primary diagnosis (ICD-10 code and description)	Number (%)*
F0–F09 – Organic, including symptomatic, mental disorders	6414 (8.0)
F10–F19 – Mental and Behavioural disorders due to psychoactive substance use	7749 (9.7)
F20–F29 – Schizophrenia, schizotypal and delusional disorders	8158 (10.2)
F30–F39 – Mood (affective) disorders	12,756 (16.0)
F40–F48 – Neurotic, stress-related and somatoform disorders	7105 (8.9)
F50–F59 – Behavioural syndromes associated with physiological disturbances and physical factors	1504 (1.9)
F60–F69 – Disorders of adult personality and behaviour	1291 (1.6)
F70–F79 – Mental Retardation	942 (1.2)
F80–F89 – Disorders of psychological development	1541 (1.9)
F90–F98 – Behavioural and emotional disorders with onset usually occurring in childhood and adolescence	3796 (4.8)
F99 – Unspecified mental disorder	4570 (5.7)
No Axis 1 diagnosis	4507 (5.6)
G – Diseases of the nervous system	304 (0.4)
Other illness codes (A-E, H-Q)	216 (0.3)
R – Symptoms, signs and abnormal clinical and laboratory findings, not elsewhere classified	231 (0.3)
S-Y – Injury, poisoning and external causes	103 (0.1)
Z – Factors influencing health status and contact with health services	18,704 (23.4)

*Total cases with primary diagnosis recorded 79,891

### Patient referral and discharge

**Figure 3:**
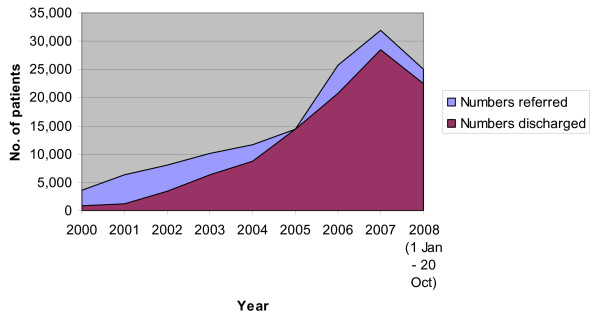
**Graph illustrating those recorded in the case register, to have been referred to or discharged at least once each year from SLAM services**.

Figure 3 illustrates data on referrals and discharges by year for SLAM patients. The number of patients that had been referred at least once over the period January 2008 until 20^th ^Oct 2008 was 25,077. Over the same period, the number of those that were discharged at least once from SLaM services was 22,538. The rise in numbers of those referred and discharged over the years is more likely to represent the growth in the use of electronic records, than actual increases in referrals and discharges. Patients are not removed from the register when discharged but no further record of their activities is kept until they access services.

## Discussion

The psychiatric case register presents a multitude of research and service development possibilities, from generation of descriptive data on mental disorder prevalence or service case mix, to applications as a case-finding tool for trials and surveys. Groups of patients that share social, demographic, clinical or environmental characteristics, or any combination of these, can be followed up as a cohort in order to investigate risk factors, outcomes and survival. As stated earlier, the key benefits of cohort studies using case register data are in ascertaining outcomes (e.g. persistence/recovery) in people with specific disorders, in contrast to conventional epidemiological cohort studies, which focus primarily on incidence of disorders in previously unaffected populations. The case register has both prospective and retrospective capabilities and is also the most appropriate tool to use in studying patterns of care, providing invaluable information for evaluating/auditing and planning services, as well as monitoring service use. In addition, the richness of free text data opens up diverse qualitative research possibilities (e.g. analyses of changing practices of medical case note keeping, or of how particular symptoms – e.g. hallucinations – are inflected by culture, age and/or gender).

The SLAM BRC Case Register is a more evolved entity than its predecessors in that it automatically updates information and maintains itself, avoiding substantial costs in time and resources. It also automatically cleans data by analysing records against patient name, date of birth and NHS number to ensure there are no duplicated records, a problem that older registers struggled to resolve. Unlike previous registers, the SLAM BRC Case Register has, in theory, an unlimited scaleable capacity. A further advantage is that, in the UK context, in contrast to most other nations, the vast majority of mental healthcare is provided by specific state-run services with minimal use of privately funded care. In this respect, SLAM has a near 100% clinical coverage in its four boroughs, which allows reasonable estimation of the total population 'at risk' from UK Census statistics. Missing people in numerator samples will be principally those choosing to access or purchase mental healthcare elsewhere. Although we are not aware of any local data enumerating this, in a UK context it is quite rare although clearly associated with income levels and a potential source of bias. As with all case registers, there are problems in the stability of diagnoses which, clinically, are subject to change based on varying presenting symptoms. Because changes on the PJS source database automatically create an archive of the original data, the BRC case register allows the researcher to view the history of diagnoses a particular patient has been given since first presentation. This enables investigation into this clinical artefact, similar to that carried out by Munk-Jorgensen and colleagues [11].

Key limitations in the case register approach are that the data available, and their quality, depend upon clinicians' accuracy and timeliness in reporting of clinical information. This is particularly pertinent to the SLAM BRC Case Register, which draws data directly from routine administrative and clinical sources (rather than the older generation of registers which have been more involved in the collection and cleaning of incoming data). Limitations on any research based around clinical services are that the availability of resources within certain areas, and cultural attitudes to using services, will affect the rate at which people access services and therefore are represented in the dataset. Case register information is limited to people who have sought help and have accessed services and does not include undiagnosed mentally ill individuals within the community. All of these issues point to the importance of close liaison between researchers using the case register and members of clinical teams who will have more familiarity in the quality of individual variables, as well as a sense of the penetrance of a given disorder and the likely representativeness of those known to clinical services. The intention of the SLAM BRC is to put in place structures and working practices which facilitate this liaison.

We believe that the SLAM BRC Case Register described here represents a 'new generation' of this research tool, drawing on very recent developments primarily in computer science and information technology but also set in the context of current debate around complex issues of autonomy, data protection and participation in health research. While the Register is now operational, a number of further developments are envisaged, particularly involving links with other databases, both within the Trust and BRC (such as, with consent, research project data and large neuroimaging and 'omics [genomics, proteomics, transcriptomics etc.] data resources) and, subject to appropriate approvals, external sources (such as small area level census statistics, primary care provider data, national mortality data). In theory, the Register could be used in the future for identifying potential research participants, and we are currently exploring the ethical and legal frameworks relating to this, as well as the opinions of service users.

## Conclusion

We describe the development of a psychiatric case register built around the fully electronic records of Europe's largest provider of secondary mental health care. Developments described include technical procedures and the security model. Key features of the SLAM BRC Register include the capacity to search and extract anonymised data including free text fields. We believe that the principles underlying this development are likely to be widely applicable and should revolutionise the capacity of one of psychiatry's oldest research methods.

## Abbreviations

SLAM: South London and Maudsley NHS Foundation Trust; IOP: Institute of Psychiatry (King's College London); BRC: Biomedical Research Centre; NHS: UK National Health Service; PJS: Patient Journey System; NIHR: UK Government National Institute for Health Research; PAS: Patient Administration System; MMSE: Mini-Mental State Examination; HoNOS: Health of the Nation Outcome Scales; CRIS: Case Register Interactive Search; ICD: International Classification of Diseases.

## Competing interests

All authors have either substantive or honorary contracts with the South London & Maudsley NHS Foundation Trust which owns the rights to the CRIS system described and stands to gain financially from any wider application.

## Authors' contributions

Analyses were carried out by MB and MS. The manuscript was finalised by RS and MS with substantial text contributions from MB, GP and FC, and further comments and significant input from all remaining co-authors who also oversaw the planning and development of the SLAM BRC Case Register.

## Appendix

### CRIS security model

#### Technical measures

1. Fields dedicated to recording personal identifiable information (PII) in the clinical database (PJS) cannot be directly searched in the case register (these include NHS number, Trust ID, name, address and other contact details, date of birth, and any stated contacts (e.g. relative's name and address)). The month and year of birth remain; postcode is truncated to the first three postcode digits/letters; ethnic groups are amalgamated so that rare groups do not act as identifiers.

2. The unique identifier used on the database is derived from each service user's NHS number by CRIS using an encryption process not accessible to researchers.

3. Free-text is anonymised: service users' and carers' PII that is entered within dedicated fields in PJS is then masked ('ZZZZ-ed out') every time that it appears in free-text entries.

4. All searches within CRIS are recorded and made available for audit by a CRIS administrator to monitor compliance with the security protocol.

#### Procedural measures

1. All CRIS users must have honorary contracts with the NHS Trust, carrying a duty of confidentiality should any potentially identifiable information be encountered by mistake. This contract necessitates an enhanced CRB check.

2. All CRIS users must sign electronically a confidentiality agreement whenever they log on (using unique Trust usernames and passwords).

3. The CRIS application and all data exported from CRIS are held within the Trust firewall, ensuring that these data are subject to the same rigorous security – both technical and policy-related – already in place for Trust identifiable electronic information.

4. A Research Governance Committee provides operational oversight and management of CRIS.

5. CRIS has received ethics approval for use as an anonymised database for secondary analysis.

6. CRIS was approved by the Trust Caldicott Committee and signed off by the Trust executive.

#### Engagement with stakeholders

1. The stakeholder participation theme of the BRC led in developing the procedural elements of the security model.

2. The Research Governance Committee overseeing CRIS is chaired by a member of the stakeholder participation theme (who is herself a mental health service user).

3. A communications plan has been developed to inform the entire Trust community (service users, carers, staff) of the existence, potential benefits and proposed uses of CRIS.

4. The BRC service user advisory group was consulted on the CRIS communications plan, and is regularly consulted about developments regarding CRIS.

## Pre-publication history

The pre-publication history for this paper can be accessed here:


